# Single-Cell Transcriptome Analysis as a Promising Tool to Study Pluripotent Stem Cell Reprogramming

**DOI:** 10.3390/ijms22115988

**Published:** 2021-06-01

**Authors:** Hyun Kyu Kim, Tae Won Ha, Man Ryul Lee

**Affiliations:** Soonchunhyang Institute of Medi-Bio Science (SIMS), Soon Chun Hyang University, Cheonan 31151, Korea; hyunkyu8505@naver.com (H.K.K.); htw5200@gmail.com (T.W.H.)

**Keywords:** single-cell mRNA sequencing, pluripotent stem cell, somatic cell reprogramming, induced pluripotent stem cell, heterogeneity

## Abstract

Cells are the basic units of all organisms and are involved in all vital activities, such as proliferation, differentiation, senescence, and apoptosis. A human body consists of more than 30 trillion cells generated through repeated division and differentiation from a single-cell fertilized egg in a highly organized programmatic fashion. Since the recent formation of the Human Cell Atlas consortium, establishing the Human Cell Atlas at the single-cell level has been an ongoing activity with the goal of understanding the mechanisms underlying diseases and vital cellular activities at the level of the single cell. In particular, transcriptome analysis of embryonic stem cells at the single-cell level is of great importance, as these cells are responsible for determining cell fate. Here, we review single-cell analysis techniques that have been actively used in recent years, introduce the single-cell analysis studies currently in progress in pluripotent stem cells and reprogramming, and forecast future studies.

## 1. Introduction

The human body consists of trillions of cells that actively coordinate with each other to perform various vital functions [1]. To understand these complicated interactions, one must determine the transcriptional expression dynamics in the cells constituting each tissue [2]. Human tissues are made up of a variety of cells, each of which undergoes genomic variation through thousands of differentiation and division cycles, and can therefore be quite heterogeneous. Dynamic molecular mechanisms in cells can be altered depending on the cell environment [3,4]. Therefore, cell fate may be explored by investigating mRNA expression at the single-cell level. As transcriptional expression levels can be successfully investigated by refining and amplifying mRNAs from single cells, technological advancements have occurred in rapid succession. In particular, next-generation sequencing (NGS) technology, developed in the early 2000s, enables the simultaneous analysis of tens of thousands of genes [5]. As a result, tissues or cell populations consisting of millions of cells could be feasibly studied at the single-cell level [6].

Such technological developments led to the establishment of a global consortium that initiated the Human Cell Atlas project to identify transcriptome and gene expression of all human tissues at the single-cell level and create a network atlas [7]. This single-cell transcriptome analysis project not only focused on a tissue cell atlas, but also extended to single-cell transcriptome analysis of various diseases as well as the development of a single cell fertilized egg, from differentiation to its growth into diverse tissues [8,9,10]. As single-cell analysis has important applications in stem cell research, single-cell transcriptome analysis has since been widely adopted. Analysis of single-cell transcriptomes can answer many questions regarding the characteristics of stem cells in an in vitro environment, the trajectory of cell fate during differentiation into mature somatic cells, and how gene expression induces somatic cell differentiation or somatic cell reprogramming [11,12,13].

Pluripotent stem cells have a self-renewal capacity and the ability to differentiate into the three germ layers, ectoderm, mesoderm, and endoderm. Pluripotent stem cell lines include embryonal carcinoma cells derived from teratocarcinomas, embryonic germ cells derived from germ cells, and embryonic stem cells (ESC) from the inner cell mass of the post-fertilization blastocyst stage [14]. Here, induced pluripotent stem cells (iPSC), established by introducing four transcription factors, and SCNT cells, established through somatic cell nuclear transfer, are also considered as pluripotent stem cell lines [15,16]. Various pluripotent stem cell (PSC) lines have been developed for laboratory research [17,18]. Despite being cultured in a undifferentiation environment, these lines include spontaneously differentiated cells with different cell statuses. Such heterogeneity in cell status can become a critical variable when attempting to understand the trajectory of differentiation. In terms of the development of PSC-induced therapeutics, the fact that cells with tumorigenic potential are always intermingled within a population remains a potential risk factor [19,20,21,22]. It is therefore very important to distinguish between the functional population and that with tumorigenic potential in the heterogenous PSC pool.

Until recently, transcriptome analysis of PSCs has been at the level of profiling transcriptomes based on bulk NGS data [23,24]. Such bulk transcriptomics has limitations regarding analysis of the heterogeneous characteristics of PSCs. Therefore, to cluster pluripotency-regulating factors and the unique expression status of differentiation potential, it is necessary to understand how different cells constitute PSC, through single-cell mRNA sequencing (scRNA-seq), and what are the interactions at the single-cell transcriptome level. Based on these data, we can understand pluripotency, simulate the development process in vitro, establish patient-customized reprogramming of stem cells, and identify the causes of genetic disorders. In this review, recent studies using single-cell transcriptomics on PSCs and factors determining cell fate since the reprogramming process are discussed.

## 2. The Single-Cell mRNA Sequencing (scRNA-Seq) Technique

**Figure 1:**
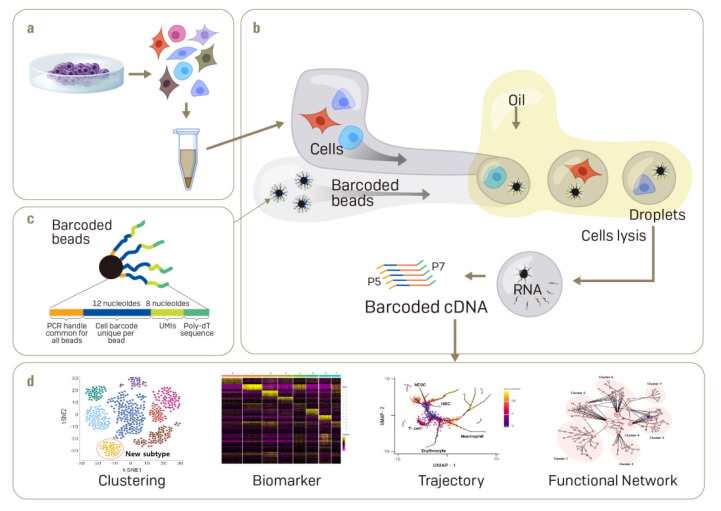
Generation of single-cell transcriptomic data using microfluidic technology. Overview of the workflow for single-cell transcriptomic analysis using microfluidics. (**a**) Cultured stem cells are initially dissociated enzymatically to generating live single cells. (**b**) Overview of the droplet-based microfluidic system. Individual cells are encapsulated in an oil droplet with a barcoded bead and captured cells are lysed within the droplets. (**c**) Microbeads coated with DNA probes that comprise a PCR handle, cell barcode, unique molecular identifiers, and poly-dT sequence. (**d**) Sequencing of cDNA yields the library of transcriptomes from individual cells, counted as unique reads per gene, and analyzed/visualized.

Various scRNA-seq techniques have been developed to date [25,26,27,28]. Since the initial introduction of platforms by different companies, the use of scRNA-seq has become commonplace [29,30]. There are three main steps for obtaining useful information through single-cell transcriptome analysis. The first step is to dissociate live single cells from tissues or cell lines in culture and retain them in their living status. This is relatively easy in cell culture; however, it is difficult to achieve in tissues or organoids without causing any cellular damage. Using mechanical and enzyme-based methods in parallel, single cells may be dissociated from a complex biological specimen to obtain live cells. The single cells need to be selected based on the expression of specific membrane proteins using fluorescence-activated cell sorting. Expert handling of single-cell dissociation or treatment can influence cell viability, which can, in turn, affect gene expression and the transcriptome (Figure 1a).

Once cells have been dissociated at high viability, they need to be captured as single cells. The two most commonly used methods of single-cell capture are microwell- and droplet-based [31,32]. Microwell-based single-cell capture involves the placement of cells one by one in a chip with a microwell, followed by reverse transcription and cDNA amplification. Various microwell-based protocols have been developed [33,34,35]. Regardless of the method used, the size of the cells should be uniform, and the number of cells analyzed at a single time remains limited. A typical commercial platform using microwell-based single-cell capture is Fluidigm C1 (https://www.fluidigm.com, accessed on 20 March 2021).

Microdroplet-based single-cell capture methods have been the primary drivingforce for the rise in single-cell transcriptomics. This method involves the preparation of a tiny oil droplet containing a single cell and a single oligo-dT primer-conjugated gel bead followed by capture and lysis of the cells within the droplet to generate a cDNA library [36,37]. The greatest strength of this method is the simultaneous processing of thousands of cells to identify a rare cell type in a heterogeneous cell population, and to track the gradual progress of cell fate through trajectory analysis. A typical commercial platform using microdroplet-based microfluidics is the Chromium of 10X Genomics (https://www.10xgenomics.com, accessed on 20 March 2021) (Figure 1b,c).

The second step in scRNA-seq is to reverse-transcribe mRNA selectively to synthesize a cDNA library. Even though the first step of single-cell capture is variable, the process of synthesizing a cDNA library is generally uniform. Usually, specific selection and reverse transcription of mRNA with a polyA tail are made with oligo-dT primers to obtain a cDNA library [38]. In this case, a short, unique barcode sequence is inserted into the middle of the primer [39]. The barcode is used in the sequencing step to identify the cell from which each RNA originated. Synthesizing cDNA through reverse transcription requires amplification of the genome, similar to a general PCR method. Since noise is inevitable in amplification, recently short sequences named unique molecular identifiers (UMIs) have been added to the primers [40]. The UMI is inserted into the primer to identify the original transcriptome from which the amplified cDNA originated. With the use of a UMI, the number of specific transcriptomes can be quantified more accurately by calibrating the noise accompanying the amplification process [41]. In all processes involving single-cell transcriptome analysis, the construction of a cDNA library is critical in determining the quality of the final result. Approximately, 20% of the transcriptome is synthesized as a cDNA library [42]; therefore, it is difficult to detect low-expressing mRNAs by scRNA-seq. The capture and amplification steps, therefore, aim to classify and capture live single cells one by one, thereby creating a high quality library. If damaged cells are captured, or if a low quality library is obtained, the result can be low total read counts, few expressed genes, and a high fraction of mitochondrial genes. If dead cells with damaged cell membranes are captured, the mRNAs are already leaking out and the total read counts would be reduced significantly; genes in mitochondria leak to a relatively lesser extent, and the proportion of mitochondrial genes among the total reads would be increased. A high rate of mitochondrial genes can also indicate the capture of more than two cells in a microwell or droplet [43]. If poor cell lysis occurs and the RNA is not liberated, the number of expressed genes would sharply decrease. If this were to be combined with results from normal cells, accurate analysis would be difficult. Therefore, the process of selecting normal cells is important for the analysis.

The third step in scRNA-seq is to sequence and analyze the cDNA library obtained from the single cells. A small amount of synthesized cDNA is additionally amplified by conventional PCR and then sequenced using a commercial sequencing platform [42]. The library is sequenced at the 3′-end. Genetic identity is determined by the approximately 100 bp adjacent to the polyA tail [44]. For this reason, the use of such sequencing in scRNA-seq is limited. To overcome this limitation, 5ʹ-RNA sequencing has been used more recently [45]. In this case, the oligo-dT primer and polyA tail are used in the soluble form, and barcodes and UMI sequences are attached to the 5′-oligonucleotide [45]. In this strategy, much more transcript sequence information can be obtained, and the bias generated in PCR analysis is effectively removed, thereby improving accuracy.

After QC, the data must be normalized since, as with bulk RNA-seq, scRNA-seq may have different total read counts per cell. As in bulk RNA-seq, the total read count per cell is divided by fragments per kilobase per million or transcripts per kilobase per million [46,47] to produce a library of equal size per cell. After QC and normalization, the resulting data undergo dimension reduction, clustering, visualization, and trajectory analysis to produce a fully transformed data set. Since tens of thousands of cells are captured, and thirty thousand gene expressions per cell are processed, scRNA-seq generates a large amount of data and high-dimensional information.

The information generated by scRNA-seq is too complex to easily visualize initially. Therefore, dimension reduction and projection are utilized to visualize gene expression data in as a single dot in 2D space [48]. Principal component analysis (PCA), t-Stochastic Neighbor Embedding (t-SNE), and uniform manifold approximation and projection (UMAP) are some methods utilized for dimension reduction to allow cells with similar gene expression to be clustered in the same 2D space [42]. t-SNE can be implemented with the use of Seurat of Cell Ranger pipeline (https://www.10xgenomics.com/, accessed on 25 March 2021) and R-package (https://satijalab.org/seurat/, accessed on 25 March 2021). Importantly, dimension reduction should exclude noise as much as possible, and prevent the loss of critical biological information. After dimension reduction, each cell is assigned unique coordinates, and those with similar genetic information are clustered. A classic method to apply to these linkages is the k-nearest neighbor, in order to simplify a complex shape with a set of cell points and lines generated by the connection of points [49]. By linking the nearest points, a well-connected “cell cluster” may be created, which can be used to find unique expression information in each cluster and hence annotate the cluster. Traditionally, making single-cell annotations or defining new cell clusters obscures the quantitative evaluation of cell identity based on the expression of the cell type-specific genes previously used. SingleCellNet, which implements a Random Forest classifier that learns cell-specific gene pairs from cross-platform and cross-species data sets and thus quantitatively evaluates cell identity at a single cellular resolution, has been developed to solve these problems and to analyze them by increasing sensitivity and specificity [50]. Thus, it becomes possible to simplify high-dimensional information and find the heterogeneity and subpopulations of a cell population [48] (Figure 1d).

## 3. scRNA-Seq in PSCs

ESCs established from the inner cell mass (ICM) of the blastocyst are PSCs that have the potential to differentiate into a diverse array of cells constituting the human body through infinite rounds of self-renewal in vitro [51,52,53]. These ESCs have drawn much attention as a critical resource, not only in developmental biology, but also in regenerative medicine [54]. An in vitro fertilized egg reaches the blastocyst stage, consisting of an ICM and trophoblast, through multiple divisions and differentiation. Generally, a human blastocyst is made up of approximately 100–150 cells. After rapid cell division, blastocysts are differentiated into a three germ layer by diverse signal transduction pathways. Special culture conditions proposed by Thomson et al. during the first establishment of human ESCs in 1998 were designed to allow for infinite self-renewal [51]. Repeated proliferation, however, leads to genomic variation and spontaneously differentiated cell populations; thus, ESCs are ultimately cultured into a heterogeneous population [55,56]. Heterogeneity among in vitro cultured PSCs can be a major obstacle when investigating early developmental stages or for developing a cell therapy product. Conventional bulk RNA-seq is limited not only in differentiating the heterogeneity of PSCs or finding small subpopulation cells, but also in distinguishing the intermingled tumorigenic populations. To define cell clusters that help explain the characteristics of PSCs and have a high utility as a cell therapy product, it is necessary to conduct scRNA-seq to profile transcriptomes at the single-cell level. Through this analysis, the heterogeneity of ESCs may be characterized efficiently.

### 3.1. scRNA-Seq in Undifferentiated PSCs

Since the publication of scRNA-seq analysis of oocytes in 2009, a variety of cells, tissues, and pathologic tissues have been analyzed in this manner. The use of scRNA-seq on human ESCs has been performed in great detail since the advent of Smart-Seq in 2012 [57]. Based on scRNA-seq analysis, hESCs were divided into eight cell clusters and modules of co-regulated genes could be analyzed [58]. In addition, using human preimplantation embryos and ESCs, the first long non-coding RNA (lncRNA) expression maps were drawn [59]. This was a significant achievement as the first single-cell transcriptome analysis conducted using hESCs. However, the Smart-Seq technique used in the study had a high possibility of error owing to sensitivity to enzyme activity and was limited in its capacity to distinguish between subpopulations, owing to its use of a small number of cells. Therefore, development of microwell- and droplet-based scRNA-seq have played a decisive role in single-cell research on ESCs [60,61]. Studies on the developmental status and heterogeneity of undifferentiated ESCs and induced pluripotent stem cells (iPSCs), using microwell- and droplet-based scRNA-seq, will be discussed below.

Using a microwell-based microfluidic chip, Messmer et al. identified transcriptional heterogeneity in hESCs [62]. Generally, pluripotency status is divided into naïve and primed states. Although both human and mouse ESCs are derived from the ICM of a preimplantation blastocyst, they have different transcriptomic, epigenetic, and morphological characteristics. Mouse ESCs are naïve cells that have a core pluripotency network, including *OCT4*, *KLF4*, and *DPPA3*. Unlike primed human ESCs, mouse ESCs have two X chromosomes activated in female mice and feature global DNA hypomethylation and dome-shaped mESC colonies [63]. However, during development, primed hESCs are considered pluripotent in the epiblast after implantation. Messmer et al. converted primed hESCs into naïve hESCs by chemical means and analyzed pluripotent status-based characteristics through scRNA-seq and compared cell subpopulations. Through scRNA-seq analysis, the researchers discovered that similar levels of pluripotency-specific markers, namely *OCT4, SOX2*, and *NANOG*, were expressed in both naïve and primed cells and that the cell population expressed many naïve *(KLF17, DPPA5, DNMT3L, GATA6, TBX3, IL6ST, DPPA3*, and *KLF5*) and primed markers (*CD24, ZIC2*, and *SFRP2*). Primed cells were shown to express the markers *HMX2* for tissue generation and *SOX11* for nerve development, showing characteristics of the late development stage. In contrast, naïve cells expressed markers related to reproductive cell functions (*HORMAD1* and *KHD3CL*) [64]. Moreover, a subpopulation of naïve hESCs that showed characteristics of both naive and primed states was identified. Such subpopulations were suggested to show formative pluripotency [65], in which cells acquire differentiation competency and are marked by the expression of early post-implantation factors such as *OTX2, SOX3*, and *POU3F1*, along with the transient loss of *NANOG* expression.

Nguyen et al. used scRNA-seq to reveal that iPSCs were heterogeneous at the transcriptional level [66]. Using 165 unique genes representing pluripotency, four independent iPSC subpopulations were identified: core pluripotent, proliferative, early primed for differentiation, and late primed for differentiation. The researchers then identified the trajectory of interactions across the populations. Although the study was limited to one iPSC line, it offered a wealth of transcriptional profiling for undifferentiated iPSCs at the single-cell level and improved the understanding of complexity and heterogeneity of iPSCs.

### 3.2. scRNA-Seq in Somatic Cell Reprogramming

The application of four defined transcription factors (*OCT4, SOX2, KLF4*, and *CMYC*) to somatic cells resulted in reprogramming to PSCs [15,67]. Since this discovery, many research groups have made significant strides in understanding transcriptional and epigenetic changes in these reprogrammed cells [68]. To reveal the molecular mechanism underlying this complex reprogramming, reprogrammed cell transcriptomes have been analyzed by NGS methods [69,70,71,72]. Through bulk NGS analysis, cells early in the reprogramming process showed changes in proliferation, metabolism, and cytoskeletal organization, whereas cells late in the reprogramming process showed activation of a pluripotent network at the global level [69,70,71,73,74]. Even if reprogramming-induced transcription factors were ectopically expressed, reprogramming efficiency was extremely low, and cells that were successfully reprogramming continued to remain intermingled with those that were not. For this reason, bulk NGS analysis is insufficient in understanding reprogramming. Not all reprogrammed cells undergo the reprogramming process at the same time or in the same sequence [75,76]. Even in the case of fully reprogrammed iPSCs, variable kinetics within heterogeneous populations suggested that defining the reprogramming checkpoints might still be possible through scRNA-seq [13].

Jaenisch’s research group was the first to analyze single-cell transcriptomes chronologically during reprogramming using a microwell-based microfluidic chip [77]. Using the C1 Fluidigm commercial platform, the researchers reprogrammed mouse somatic cells, quantitatively analyzed 48 genes in hundreds of cells that acquired pluripotency, and identified cell cycle regulatory genes and activation factors that promoted reprogramming. The expression of *ESRRB, UTF1, LIN28*, and *DPPA2* was a better predictive factor than *FBXO15, FGF4*, and *OCT4*, which had previously been proposed as reprogramming markers. In particular, after activation of pluripotency genes, reprogramming was observed to occur in a hierarchical manner. Activation of endogenous *SOX2* was considered to be an event occurring upstream prior to full acquisition of pluripotency. Using scRNA-seq, the molecular determinants for induction of epigenetic changes in early stochastic and late deterministic phases were determined through a hierarchical model, transcriptome analysis, and late-reprogramming marker analysis.

Lin et al. performed scRNA-seq using Fluidigm C1 while focusing on three transcription factors involved in conventional and chemical reprogramming [78]. During reprogramming, the cell population became heterogeneous owing to stochastic cell fate determination. Using new mathematical algorithms for single-cell reprogramming analysis, the cells were classified into categories of reprogramming and non-reprogramming potential. In addition, an accurate branch point was obtained by dividing into reprogramming potential and non-reprogramming potential clusters through scRNA seq results, which provided more detailed information on the correlation of the genes involved at a specific time. The existing buck-mRNA analysis reported interferon signaling and innate immunity promote reprogramming [79,80], but suggested that INF-gamma act as a barrier in the last stage of cell fate conversion. The scRNA seq study not only provides a high resolution information and the landscape of existing reprogramming, but also suggests the existence of a barrier during reprogramming, which is significant in that it provides a clue to overcoming the limitations of existing reprogramming.

Another strength of scRNA-seq analysis in reprogramming research is the ability to draw insights related to the direction of the reprogramming trajectory. Bulk RNA-seq-based reprogramming research only captures snapshots of cells during reprogramming and does not provide information on dynamic processes; therefore, it can be difficult to explain the trajectory of reprogramming cells. By collecting data at multiple time points throughout the reprogramming process, a reprogramming trajectory may be proposed [12]. To this end, researchers performed scRNA-seq on reprogrammed mouse iPSCs at 12 h intervals to determine cell fate. The reprogramming environment was reconfigured in 315,000 scRNA-seq profiles, and the Waddington-optimal-transport algorithm was applied to create a reprogramming trajectory, a development program wider than any previously obtained. Cells were found to transition from mesenchymal to epithelial; populations related to pluripotency, extra-embryo, and nerve cells were generated; and each population included multiple micro-subpopulations. Through their scRNA-seq analysis, reprogramming waves appear several times when somatic cell reprogramming occurs, and Obox6 is revealed as a transcription factor that appears after the second reprogramming wave. Evaluating interaction scores for ligands in stromal cells with receptors expressed in iPSCs, the authors found a paracrine factor, *GDF9*, that mediates intercellular interactions when reprogramming takes place, confirming that it can increase reprogramming efficiency. The study showed that transcription factors such as Obox6 and the paracrine signal *GDF9* can affect cell fate transitions, increasing reprogramming efficiency [81].

Reprogramming is a stochastic process. A very small number of cells in a reprogramming-induced population are actually reprogrammed [82]. Therefore, it is difficult to detect changes in fully reprogrammed iPSCs. Hence, analysis of single-cell transcriptomes is essential to accurately understand the entire reprogramming process in somatic cells, to identify cell fate determinants, and to develop strategies for overcoming the reprogramming barrier. Tran et al. conducted an analysis using an average of approximately 55,000 reads and 13,000 uniquely identified transcripts per cell, which corresponded to a total of 18,005 genes detected across all cells [13]. At the time of reprogramming from mouse embryonic fibroblasts (MEFs) to iPSCs, the researchers identified the path, speed, and efficiency of cell fate conversion of the reprogrammed cells by determining pluripotency of 14 cell clusters in a time-course analysis. At that time, they observed the expression pattern of four genes and found that all of the mesenchymal-related genes are not downgraded at the same stage, and the cell cycle and antihypertensive genes are temporarily controlled. Downregulation of mesenchymal genes and upregulation of *CDH1* were found to be independently regulated, whereas co-expression *NANOG*, *SALL4, TDGF1*, and *EPCAM* within the same cell predicted a more homogenous transition to an iPSC state. The process of transition to iPSC meant a continuous change in cell fate. If a cell population is very heterogeneous, it is impossible to understand the reprogramming by only bulk sequence analysis of cells at the end points [13]. Based on the review of multiple theses, scRNA-seq helped reveal that PSCs were heterogeneous, that there were multiple cell fate transition branch points in the reprogramming process, and that the mechanism for each can be unraveled.

Xing et al. simultaneously conducted scRNA-seq and single-cell ATAC-seq (scATAC-seq) to further clarify the analysis of the entire body of a single-cell in human somatic cell reprogramming [11]. scATAC-seq is an epigenomic profiling technique for measuring chromatin accessibility and discovering cell type-specific regulatory mechanisms [83]. The combination of scRNA-seq and scATAC-seq provide more comprehensive molecular profiles of individual cells and their identities. In human somatic cells, single-cell analysis combined with scRNA-seq and scATAC-seq during reprogramming showed that the transition from a network controlled by *FOSL1* to a network controlled by *TED4* leads the cell to a pluripotent state. The combinational application of scRNA-seq and scATAC-seq to study these reprogrammed heterogeneous groups continues to advance our understanding of reprogramming efficiency-related hurdles [84].

## 4. Conclusions

Biologists have long aimed to identify the expression of genes at the single-cell level. Ten years ago, this goal could be accomplished mainly through RNA in situ hybridization, immunostaining, or FACS for specific proteins. However, in these methods, only a certain number of genes could be identified in each experiment, and bulk-mRNA sequencing for the total population had limitations owing to heterogeneity. To overcome these limitations, track heterogeneous cell subpopulations, and understand pathway dynamics, single-cell transcriptome analysis has been widely accepted since its introduction in 2009 [2,57]. Particularly in stem cell research, it can be widely applied for identifying cell differentiation potential, cell fate determinants, and heterogeneity. This review focused on undifferentiated pluripotent status and reprogramming pluripotency. Recently, single-cell transcriptome analysis has helped induce undifferentiated stem cells in vitro and enable their differentiation into diverse cells, generating a “pseudo-time” trajectory of the fate determinants of stem cells [11,12,79]. Studies using scRNA-seq have helped identify functional cellular subpopulations, enhance developmental studies, and contribute to identifying causes underlying diseases. Despite considerable development over the last ten years, there remain limitations in the detection of mutations in undetected cell populations owing to 3′-mRNA sequencing [66]. Although scRNA-seq enables identification of markers of cell populations and subpopulations, it cannot yet offer sufficient information on the interactions across subpopulations and organisms. If a technique for analyzing proteomes and metabolomes at the single-cell level can be developed, along with development of methods to combine these large data sets, safer and more efficient stem cell therapy products may be designed therefrom.
